# Microbial Markers Profile in Anaerobic Mars Analogue Environments Using the LDChip (Life Detector Chip) Antibody Microarray Core of the SOLID (Signs of Life Detector) Platform

**DOI:** 10.3390/microorganisms7090365

**Published:** 2019-09-18

**Authors:** Laura García-Descalzo, Victorino Parro, Miriam García-Villadangos, Charles S. Cockell, Christine Moissl-Eichinger, Alex Perras, Petra Rettberg, Kristina Beblo-Vranesevic, Maria Bohmeier, Elke Rabbow, Frances Westall, Frederik Gaboyer, Ricardo Amils, Moustafa Malki, Viggo Marteinsson, Pauline Vannier, Pascale Ehrenfreund, Euan Monaghan, Andreas Riedo, Patricia Cabezas, Nicolas Walter, Felipe Gómez Gómez

**Affiliations:** 1 Centro de Astrobiología (CSIC-INTA), 28850 Madrid, Spain; parrov@cab.inta-csic.es (V.P.); villadangosgm@cab.inta-csic.es (M.G.-V.); ramils@cbm.uam.es (R.A.);; 2 UK Center for Astrobiology, School of Physics and Astronomy, University of Edinburgh, Edinburgh EH9 3FD, UK; c.s.cockell@ed.ac.uk; 3 Department of Internal Medicine, Medical University of Graz, 8036 Graz, Austria; christine.moissl-eichinger@medunigraz.at (C.M.-E.); akperras@gmail.com (A.P.); 4 BioTechMed Graz, 8010 Graz, Austria; 5 Department of Microbiology and Archaea, University of Regensburg, 93040 Regensburg, Germany; 6 German Aerospace Center (DLR), Research Group ‘Astrobiology’, Radiation Biology Department, Institute of Aerospace Medicine, 51147 Cologne, Germany; Petra.Rettberg@dlr.de (P.R.); Kristina.Beblo@dlr.de (K.B.-V.); Maria.Bohmeier@dlr.de (M.B.); Elke.Rabbow@dlr.de (E.R.); 7 Centre de Biophysique Moléculaire, Centre National de la Recherche Scientifique (CNRS), 45100 Orléans, France; frances.westall@cnrs-orleans.fr (F.W.); frederic.gaboyer@cnrs-orleans.fr (F.G.); 8 Centro de Biología Molecular Severo Ochoa (CSIC-UAM), 28049 Madrid, Spain; mkasma@hotmail.com; 9 MATIS-Prokaria, 113 Reykjavík, Iceland; viggo@matis.is (V.M.); pauline.vannier@matis.is (P.V.); 10 Faculty of Food Science and Nutrition, University of Iceland, 101 Reykjavík, Iceland; 11 Leiden Observatory, Universiteit Leiden, 2300 Leiden, The Netherlands; p.ehrenfreund@chem.leidenuniv.nl (P.E.); monaghan@strw.leidenuniv.nl (E.M.); riedo@strw.leidenuniv.nl (A.R.); 12 European Science Foundation (ESF), 67080 Strasbourg, France; PCabezas@esf.org (P.C.); nwalter@esf.org (N.W.)

**Keywords:** anaerobic environments, Mars, analogues, biomarkers, microarray

## Abstract

One of the main objectives for astrobiology is to unravel and explore the habitability of environments beyond Earth, paying special attention to Mars. If the combined environmental stress factors on Mars are compatible with life or if they were less harsh in the past, to investigate the traces of past or present life is critical to understand its potential habitability. Essential for this research is the characterization of Mars analogue environments on Earth through the development of techniques for biomarker detection in them. Biosensing techniques based on fluorescence sandwich microarray immunoassays (FSMI) have shown to be a powerful tool to detect biosignatures and depict the microbial profiles of different environments. In this study, we described the microbial biomarker profile of five anoxic Mars analogues sites using the Life Detector Chip (LDChip), an antibody microarray for multiple microbial marker detection. Furthermore, we contributed to new targets by developing a new 26-polyclonal antibodies microarray using crude extracts from anaerobic sampling sites, halophilic microorganisms, and anaerobic isolates obtained in the framework of the European Mars Analogues for Space Exploration (MASE) project. The new subset of antibodies was characterized and implemented into a microarray platform (MASE-Chip) for microbial marker searching in salty and anaerobic environments.

## 1. Introduction

Searching for life or potential habitable conditions beyond Earth is a central objective in Astrobiology [1,2,3]. The habitability of early and present Mars has been investigated extensively during the last decades [2,3,4,5,6,7,8,9,10].

At present, Mars seems to be a dry planet without liquid water on its surface, with an atmosphere mostly composed of carbon dioxide (CO_2_) and lacking a significant protecting ozone layer. Thus, the present surface of Mars is exposed to strong UV (ultraviolet) radiation with extremely low temperatures and without liquid water on its surface, although it could exist in the subsurface [7,11,12,13]. These current conditions make the Red Planet, at first glance, inhospitable for life on the surface, but conditions may have not always been that harsh. Around 4.1–3.7 billions of years ago (Ga), the planet had a thicker atmosphere and moderately stable and warmer surface temperatures, which could have allowed for the presence of liquid water on the surface [14,15]. Moreover, orbital images from Mariner 9 [16] provided the first evidence for past liquid water on Mars, and the Mars Reconnaissance Orbiter [17] showed fluvial features at an ever-increasing resolution, suggesting water flow on the surface of Mars in the past. On the other hand, there are data indicating the possibility of water on the present Mars at the equator where low-albedo features form and grow during the warmer months and disappear in the cold seasons, causing so-called recurring slope lineae (RSL), which could be explained by the existence of liquid brines near the surface; however, this is still an open question [13].

Though the source of water that could be the origin of these features remains unclear, it seems that under certain conditions, small quantities of liquid water, possibly brines, can still form close to the Martian surface under the current climate [13,18,19]. In comparably salty terrestrial environments, different microorganisms can be found [4,20,21,22,23], adapted to the high content of salt where the deliquescence of hygroscopic minerals provides liquid water available for microorganisms [4]. Therefore, from the perspective of salt content, the brines on Mars could provide aqueous, highly salty biotopes suitable for adapted halophilic microorganisms [4,21,22]. Geochemical data from Martian meteorites and planetary exploration instruments such as the high oxidant (iron-rich smectite clays, iron oxides, and magnesium sulfate) composition of the surface [24,25,26,27] and the cold desiccated radiation-bathed surface of the planet indicate that habitable conditions are most likely restricted to the subsurface rather than on the surface [8,28], where biota, which may have inhabited Mars in the past, were forced to retreat and might still persist today [7]. This retreat could have induced microbial life, if it ever occurred, likely to be more abundant in protective niches far from radiation, desiccation (in brines) and lack of oxygen. This drove us to study the microbial ecology of anoxic analogue environments on Earth, which had been less well investigated up to now, even taking into account that most known extraterrestrial environments are oxygen-free or contain very low abundances of this gas. Though recent studies [29] have suggested that in certain Mars areas, enough oxygen may be available for some microbes to breathe, it is expected that the predominant type of potential organisms would be anaerobes. Precisely, the thin Martian atmosphere contains 0.14% oxygen, and the surface and subsurface of that planet are prone to be suitable for facultative anaerobic and the majority of strict anaerobic microorganisms [30].

The anoxic analogous environments selected show a number of characteristic parameters, such as (i) low water activity, (ii) low temperature, (iii) the restricted availability of (complex organic) nutrients, (iv) oxygen limitation, and other stress factors for microbial life (e.g., high salinity, high acidity, and radiation) [31]. Since the greatest challenge would be to find living organisms on Mars, the identification of biomarkers from physico–chemical and metabolism processes is a plausible approach. The study of the detection and preservation of microbial biosignatures in anoxic analogous extreme terrestrial environments will broaden our knowledge and improve strategies for the search for potential microbial traces in protective habitats on Mars.

In the framework of MASE (Mars Analogues for Space Exploration), a European Seventh Framework Programme, FP7-funded project, we investigated several anoxic Mars analogues sites, including (i) cold sulfidic springs, (ii) an acidic lake environment, (iii) a hypersaline subsurface environment, and (iv) rock glacier sites in order to obtain a collection of different anoxic samples (soil, water and sediments) that were subjected to a set of investigations. Throughout these investigations by the MASE project team, the whole anaerobic community was characterized by sequencing [31], culture enrichment, and the isolation of microbial strains, which were deposited in the German Collection of Microorganisms and Cell Cultures GmbH (DSMZ, Germany) for long-term storage and distribution to other interested scientists [30]. Studies of physiology, biochemistry, several stress resistance assays, the mineralization of extremotolerant strains from these analog sites and metabolomics studies [32,33,34,35] were carried out, as well as the subject of this paper: The search for detectable biosignatures and the description of analogous sites’ biomarker profiles.

In the last two decades, several works have proposed immunosensor techniques based on bioaffinity through the use of antibodies for in situ analysis for biomarker detection in planetary exploration [36,37,38,39]. One of the most promising analytical methods among them is the Signs of Life Detector (SOLID)-LDChip system [36,38,40] based on a multiplex fluorescence sandwich microarray immunoassay (FSMI), designed for and previously applied in astrobiology studies as well as in microbial ecology studies [40,41,42,43]. The LDChip is an antibody microarray-based biosensor for monitoring the presence of microbes and their metabolic products in different kinds of environmental samples. It was developed and implemented for in situ detection of biomarkers and it is the core sensing device of the SOLID instrument [40]. The LDChip has been used for detecting prokaryotes’ biomarker profiling in different extreme environments, which include the acidic iron-rich sediments of Rio Tinto in Spain [44,45,46], subsurface sediments cores in the hypersaline Atacama Desert [40], the surface and sediments drilled on Deception Island in Antarctica [47] and Andean glacial lakes [43,48].

In this article, we report the heterogeneity of the microbial profiles of a whole set of samples from the Mars analogues characterized by MASE team by using the LDChip. Additionally, and as a direct output of MASE microbial isolates, this study contributes with a new set of microbial targets and antibodies from anaerobic and halophiles microorganisms for biomarker detection. This can be used to implement a new LDChip for planetary exploration. The new LDChip proposed here (MASE-Chip) consists of 26-polyclonal antibodies against the mentioned halophiles and MASE anaerobic isolates.

## 2. Materials and Methods

### 2.1. Mars Analogues Selection and Sampling

Since none of the terrestrial Mars analogue environments possess all Martian stress factors at once and in the same location, the selected MASE sites—taken in combination—are a good representative of not every but a desirable range of conditions on Mars throughout its history [30]. The MASE sites share some characteristics that were used as criterion for their selection: (i) They are limited in nutrients, (ii) anoxic in nature, and (iii) correspond to a particular postulated environment representative of past or present-day Mars. The MASE sites where sampling was carried out are the following:

- Boulby mine in the United Kingdom (UK), a potash mine one kilometer deep in the north-east of the UK that contains sequences of Permian halite and sulfate deposits [49] with anoxic conditions inside [50]. The physicochemical characteristics of ponds in Boulby (salt-saturated, pH neutral, subsurface bodies of water that host anoxic environments in their sediments) provide an environmental analogue to potential water bodies that may have existed in Martian evaporitic water [51,52].

- Grænavatn Lake in Iceland, one of several lakes in the geothermal area of Krýsuvík that originated from volcanic explosion craters [53]. The near-lakeshore environment is characterized by periodic desiccation and an acidic pH, with volcanic rock-water interactions and low water temperatures. This makes it a Mars analogue of deltaic deposits found, for example, at Jezero Crater, which closely resemble those found in terrestrial lacustrine settings [54,55].

- Regensburg in Germany, where many anoxic sulfide-containing springs emanate from the subsurface. Two of them have previously been studied at the Sippenauer Moor and Islinger Mühlbach areas [56,57,58]. Both are characterized by a main sulfidic spring that rises into a streamlet where whitish mats of sulfide-oxidizing bacteria cover the submerged surfaces. These aquifers are very similar to sulfidic cave springs that are rich in sulfide, ammonia and sulfate but poor in dissolved organic carbon. The Sippenauer Moor and Islinger Mühlbach sites are independent, and they are not connected in the deep subsurface, even though they both emanate from Mesozoic sediments formation and are fed by the same deep groundwater flow within the pre-alpine Tertiary Molasse basin [59,60]. The domination of the sulfur cycle on Mars [61] makes these aqueous environments that contain diverse sulfur species useful potential analogues.

- Kaunertal, Austria: The edges and bedrock of glacial environments offer the possibility of collecting samples from perennially frozen soils and ices. Glacier samples were collected from a rock glacier in the Kaunertal, Austria, in the frame of the Austrian Space Forum’s AMADEE-15 analog program. The Kaunertal valley is 28 km in length and runs southeast from the town of Prutz to the Kaunertal Glacier. Samples of soil were taken from the glacier and from a streamlet derived from melted glacier ice [30].

In summary, sediments and water were sampled from all MASE sites. In Boulby (hypersaline environment), two different brine seeps were sampled at the Billingham Bath location (samples referred to as Bou.I and Bou.II hereafter; coordinates: 54.5575; −0.8202 in mine surface). These sites were located ~14 km distance from the coastline under the North Sea. Regarding sulfidic springs (Regensburg), samples were taken at two different sampling locations in Regensburg at Sippenauer Moor (SM) (sulfidic spring SM, coordinates 48.8685; 1.9563) (water (SMw) and sediment (SMs) samples), and at Mühlbacher Schwefelquelle Isling (MSI) (sulfidic spring MSI, sediment sample, coordinates: 48.9857; 12.1272). In Iceland, samples were extracted in anoxic conditions at two different sampling sites at Grænavatn Lake (Icelandic acidic lake) (IS.SS1, coordinates 63.8857; −22.0544 and IS.SS3, coordinates 63.8830; −22.0565). Finally, Glacier samples were collected from a rock glacier in the Kaunertal, Austria (Glacier) (samples named G. ss1 I, G. ss1 II, G. ss2 I, G. ss2 II according to sample sites 1 or 2 and duplicates in each site, I and II).

To ensure sampling in anoxic conditions, the field equipment included, among others, Duran glass bottles medium sealed with a rubber bung and a screw cap containing anoxic atmosphere. As reducing agents, aliquots of filter sterilized cysteine-HCl (50 mL of 2.5% w/v) were used to adjust the anoxic atmosphere, and resazurin (20 mL of 0.1% w/v) was used as a redox-indicator. Syringes, cannulas, wipes soaked in ethanol and other components were used for the sampling. The detailed list and procedure that could be broadened in [30] included previous steps to prepare the sampling anaerobic glass bottles as follows: 0.5 mL of a resazurin solution (a redox-sensitive dye to monitor redox potential) were pipetted into each 100 mL Duran laboratory bottle. Subsequently, the bottles were closed with a butyl rubber stopper, which was held in place with an appropriate screw cap. This step was followed by three gas-vacuum exchange cycles using nitrogen in order to obtain an oxygen-free atmosphere within the bottle. The last step included purging with nitrogen until an overpressure of 0.2–0.5 bar was reached. Once this was done, the bottles were autoclaved for 40 min at 121 °C.

The sampling procedures followed the subsequent steps: Prior to the removal of the screw cap and the rubber stopper, the overpressure within the anaerobic Duran glass bottle was removed by the use of a sterile needle. The butyl rubber septum was sterilized using ethanol. The sediment and water samples were taken the corresponding pool in each case as deeply as possible (approximately 30–40 cm below the water level in Boulby and Grᴂnvtan using a sterile Falcon tube that was attached to the end of a pole and 20–30 cm below the water level in the sulfur springs of Regensburg using anaerobic glass bottles). Once the rubber stopper was aseptically removed, the sediment was immediately poured into the glass bottle and filled up to the brim with site water that was also collected. After filling the bottles with sediment, brine or water, they were closed with the rubber stopper. The last step included the addition of the reducing agent mentioned (1 mL cysteine-HCl of a 2.5% solution) to depress and poise the redox potential at optimum levels. In every moment, the highest care was taken to keep samples away from contamination and to minimize oxygen, possible traces of which were quickly removed by the methods indicated before.

Samples were taken in three replicates of 100 mL bottles to distribute them among the team for each purpose and stored at 4 °C until their use.

### 2.2. Immunoprofiles of MASE Sites Using Fluorescence Sandwich Microarray Immunoassays

**Figure 1:**
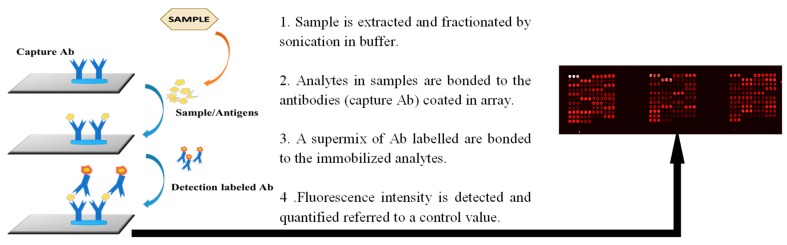
The scheme of the sandwich immunoreaction occurring in each spot printed in the microarray with the main steps of the procedure. The image on the right is an example of one of the nine arrays in the slide. Red spots (printed in triplicates) indicate the recognition of epitopes in the sample between the antibodies fixed in the slide and labelled antibodies in the supermix.

The different samples described were analyzed by a sandwich microarray immunoassay through a 168 antibody-containing LDChip. This LDChip includes antibodies against (i) whole microbial cells representatives of the main phylogenetic groups of prokaryotes/archaea, (ii) extracellular polymers, (iii) environmental extracts from terrestrial analogues for Mars, (iv) proteins and peptides from well-conserved anaerobic metabolic pathways, (v) exopolysaccharides, and (vi) amino acids, among others biomolecules used in metabolism. The antibodies printed in the mentioned chip were performed in rabbits from samples in the list below. The immunoglobulin-IgG-fraction of each antibody from the rabbits serum was purified with protein A and spotted in triplicates over the surface of epoxy-activated glass slides (Arrayit, CA, USA) [36,62] (Table S1). Over the explained LDChip, the sandwich immunoassay with the MASE site samples (Figure 1) was carried out, adapted from the procedure described previously in [36,62]. This consisted of an ‘antibody-antigen-labelled antibody’ (Ab-Ag-Ab*) bound where the antigen comes from the extraction of epitopes from samples using 2 g (for sediment samples) or 50 mL (in the case of water samples) of each one. These epitopes were obtained by sonication of the samples (3 cycles of 30 s at 90% maximum amplitude on ice; DR. Hielscher 50W DRH-UP50H sonicator, Hielscher Ultrasonics, Berlin, Germany) in a buffer extraction (NaCl 0.3M, Tris-HCl 0.4 M pH = 8, 0.1% Tween 20) and filtered through a 5 µm nylon membrane to remove possible minerals and coarse material.

First, and prior to displaying the sample, the microarrays were blocked in two sequential steps of 2% and 5% BSA (bovine serum albumin) in a Tris-HCl buffer to minimize unspecific joints. Then, the samples were incubated over the LDChip for 1 h at room temperature to allow for interactions between the capturing antibodies (antibodies spotted in the microarray) and molecules present in the sample. This was carried out inside a MultiArray Analysis Module (MAAM), in which up to nine assays can be processed at the same time [63]. Afterwards, a washing step in which the buffer removed the excess of the unbound sample occurred. Then, the microarray was incubated for 1 h with an Alexa Fluor-labelled mixture of the same kind of antibodies (Abs) coated in its surface (reporting Abs), resulting in a sandwich conformation (Figure 1). The excess of labelled antibodies—those not bound—was washed, and the slides were scanned with a red laser (635 nm) that excited the reporting Abs.

**Figure 2:**
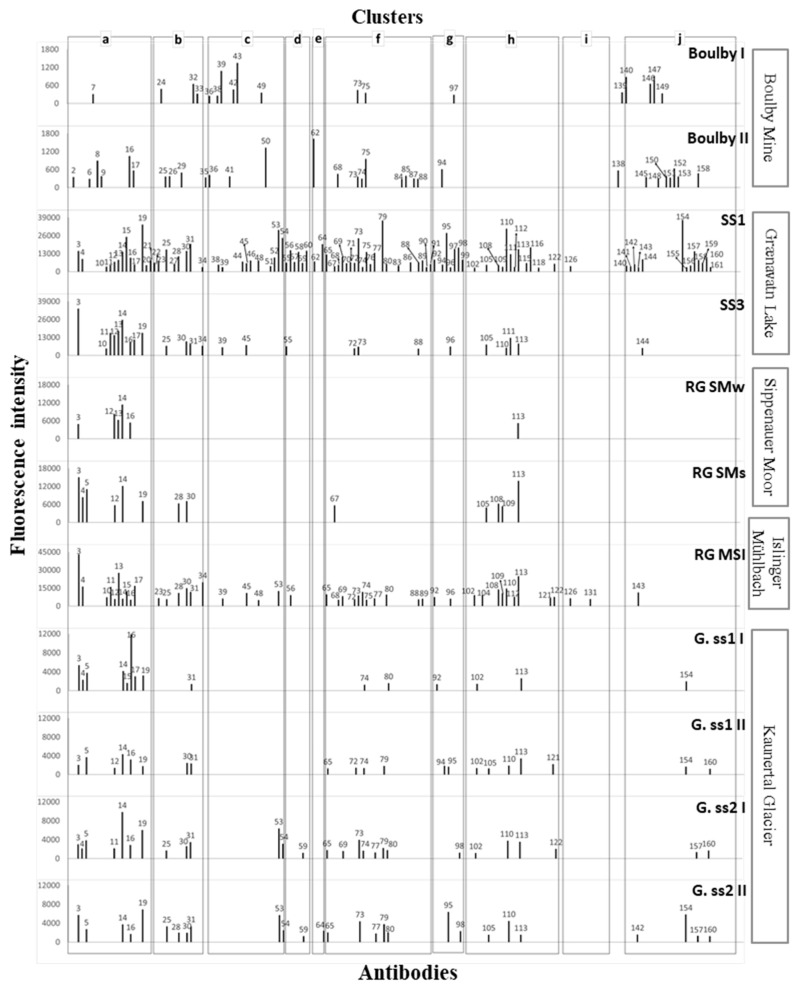
Immunograms of water and sediment samples of each Mars Analogues for Space Exploration (MASE) site (explained in Material and Methods section). Plots show the positive immunodetections and their fluorescence intensity. The peak numbers correspond to the antibody detected in each reaction and are listed in Table S1. Letters above from **a** to **j** frame those antibodies of positive immunodetections that has been clustered as follows: **a**. Fe–S oxidizers cultures; **b**. metal-acidic environment (water source); **c**. psycrophilic cultures; **d**. iron reducers; **e**. spores; **f**. mesophilic cultures; **g**. archaea; **h**. cyanobacteria; **i**. perchlorate reducers; and **j**. proteins and peptides. Moreover, some groups are represented by a sole antibody: Peaks 35, Ab ID: IIC1C1 geothermal environment; 138, Ab ID: VIID1BF, mesophilic environment; and 139, Ab ID: VD2BF, biofilm from mines. Please note that scales of the Y axes (fluorescence intensity) are not the same for every site, but they remain homogenous within each one.

The fluorescent signal can be captured by a highly sensitive CCD (charged-coupled device) detector and stored as a Flexible Image Transport System (i.e., .fits) image file that could be processed and analyzed by conventional microarray software. Thus, after the immunoassay were completed, and the slides were analyzed in the laboratory by illuminating them with 635 nm light and imaging their fluorescent emission at 650 nm with a GenePix 4100A scanner. The images were quantitatively analyzed using GenePix Pro software (Genomic Solutions). A blank was always run in parallel with only a buffer as the “antigenic” sample and then revealed with the same fluorescent antibody or antibody cocktail as the real samples. This image was used as a baseline to calculate spots intensities in the tested sample. The final fluorescence intensity (F) for each antibody spot was calculated with the equation F = (F_sample_ − F_blank_ − 2.5F_avcontrol spots_), where F is the fluorescent intensity at 635 nm minus the local background, as quantified by the software (GenePix Pro); F_sample_ is the total fluorescence signal of the sample; F_blank_ is the total fluorescence signal of the blank; and F_avcontrol spots_ is the average fluorescent signal of the control spots. These control spots are located in different parts of the microarray and consist of BSA, the buffer, and spots corresponding to the pre-immune antisera (more than 50). Because, theoretically, they should not exhibit any fluorescent signal, they were used as a baseline for fluorescence. Therefore, 2.5 times the F_avcontrol spots_ was used as a stringency cutoff to minimize false positives, taking those above this value as real positives. The spots with obvious defects (missing or very tiny spots or an artifact due to a bad wash or dust in the array) or duplicated spots whose standard deviation was 0.2 times higher than the mean were not considered for quantification [40]. The positives fluorescent signals quantified were plotted as an immunogram (Figure 2).

### 2.3. Building a Microarray from MASE Sites and Salty Environments

In this study, a 26-polyclonal antibody microarray to MASE isolated strains was developed using samples from the sampling sites described, isolated strains from these sites, and was complemented with antibodies from previous studies (Table 1).

Thus, antibodies obtained from 15 isolated microorganisms from MASE sample sites and a set of 11 antibodies available from Molecular Ecology Laboratory (Centro de Astrobiologia-CAB, Consejo Superior de Investigaciones Científicas-Instituto Nacional de Técnica Aeroespacial, CSIC-INTA, Madrid) collection of halophilic microorganisms were used to develop and build a new microarray for Mars analogue environment monitoring. Firstly, the 15 isolates representative from MASE sites were selected to immunize rabbits and obtain polyclonal antibodies (against the antigenic fraction), and these were carried out by Biomedal Company and the Immunology Department at Hospital Fundación Jimenez Díaz in Madrid. The used antigens were prepared from isolated bacteria homogenates that were washed three times in PBS by centrifugation for 10 min at 13,000 rpm and then ultrasonicated (4 cycles of 30 s at amplitude of 90%, stopping 30 s on ice between cycles) and filtered by a 5 µm membrane before being injected into rabbits. Secondly, after six weeks of immunization, the IgG fraction of the antibodies performed and recovered from rabbit serum were purified by protein A affinity columns (Sigma). Finally, the set of purified antibodies were fluorescently labelled with Alexa 647 fluorochrome at a concentration of 2 mg mL^−1^, as recommended by the provider (Molecular Probes, Invitrogen) to be used as the tracer antibody in the sandwich immunoassay. The other set of the same non-labelled antibodies were printed in a triplicate spot pattern on epoxy-activated glass slides as the capture antibodies for the samples/antigens.

In addition, control spots containing only BSA, the buffer and a serial dilution of fluorescent labelled pre-immune antiserum were also printed to be used as baselines and to subtract their average fluorescence from the sample´s readings. These fluorescence spots of pre-immune antiserum were also used as indicators of the frame of each individual array to ease the image analysis after its recording. This microarray, MASE-Chip, was printed in a 3 × 8 (3 in each row and 8 in each column of the chip surface) array setting in order to be available for 24 different assays at the same time.

### 2.4. Testing and Validating the New Antibodies and MASE-Chip

As a first step for examining the accuracy of the MASE-Chip and to set the optimal assay conditions to be used in future experiments, two types of assays were carried out with the new antibodies from the MASE isolates generated in this study. These two assays were used for testing both the more efficient concentration of each labelled antibody and the minimum concentration of antigen that could be detected by the microarray. In the first place, a fixed concentration of the antigen was used, and that of its corresponding fluorescent antibody was applied in serial dilutions (from 1/500 to 1/5000) to set the best working concentration. Once the concentration of the labelled antibody was established, the concentration of the antigen was varied until the detection limit was determined from the analysis of the images corresponding to different dilutions, providing calibration curves. Accordingly, the cross reactivity, and therefore the specificity, could be revealed, allowing us to disentangle the potential points of non-specificity of the microarray to be taken into account in further experiments. The analysis procedure to determine the positive immunodetections by the immunogens and antibodies followed the same principles described before for the generation of immunoprofiles by means of fluorescence signals over a cutoff value.

## 3. Results

### 3.1. Biomarker Profiles of MASE Sites

**Figure 3:**
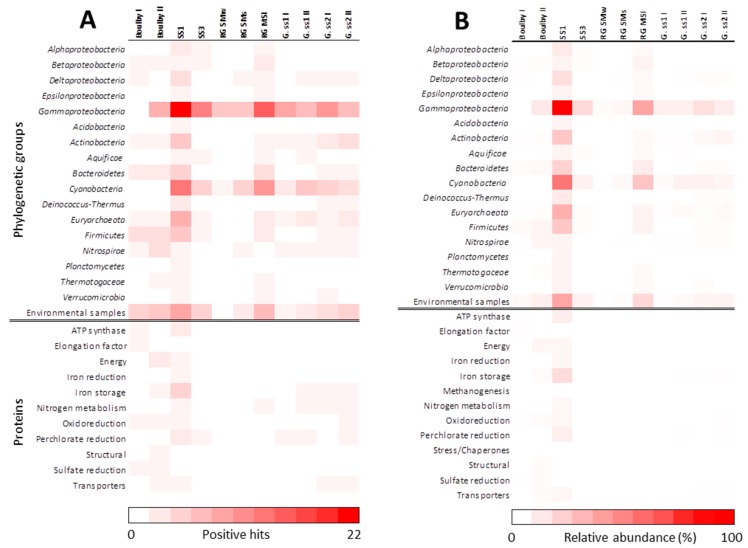
Heat map showing the results obtained with the Life Detector Chip (LDChip)168 immunoassay in samples from MASE sites (Boulby I (Bou-I) and Boulby II (Bou-II) from Boulby Mine (hypersaline environment); IS.SS1 and IS.SS3 from Iceland-Grᴂnvatn Lake (acidic lake); RG. SMw (water) and RG. SMs (sediments) from Sippenauer Moor (sulfidic spring SM); RG. MSI form Islinger Mühlbach Schewefelquelle (sulfidic spring IM); and G.ss1-I, G. ss1-II, G. ss2-I, G. ss2-II from Kaunertal Glacier, Austria (glacier). The antibodies that showed positive immunodetections were clustered by phylogeny of the targets, protein function, or environmental origin and plotted in a scale from white to red. (**A**) The number of hits is represented from 0 to 22, as the maximum number of hits of antibodies (Abs) from the same group detected in a sample. Here, certain underestimations may result from representing the positive immunodetections in those groups with a lower number of antibodies represented in the chip. In (**B**), the number of hits was normalized by the relative weight of each group represented in the chip and expressed as the relative abundance in percentage. The immunoprofiles of the samples did not change substantially with the normalization, but the intensities of detection decreased in terms of abundance. Total antibodies showing positive immunodetections appear in Table S1, marked with “+,” where the identification code of the antibody detected, their immunogenic fraction, and the sources (environmental sample or microbial strain) used for its production are also indicated.

Anaerobic water and sediment samples from each selected MASE site (Regensburg, Germany; Gænvatn Lake, Iceland; Boulby Mine, UK; and Kaunertal Glacier, Austria) were processed in the laboratory by sonication (material and methods section) to obtain the extracts or epitopes that were analyzed using the LDChip, which included different antibodies (small peptides and molecules, proteins, exopolysaccharides and cell extracts; Table S1). The fluorescent images obtained from the assay were analyzed, and the fluorescence intensity of each antibody spot on the microarray was quantified. Data from this analysis were plotted as immunograms or biomarker profiles (Figure 2). The immunograms showed several positive antigen-antibody signals in all the samples analyzed: Boulby Mine—hypersaline environment (Bou. I and II); Graenvatn Lake—acidic lake (IS.SS1 and IS.SS3); Regensburg—sulfidic springs (SMw, SMs and MSIs); and Kaunertal Glacier, Austria—glacier (G.ss1-I, G. ss1-II, G. ss2-I, G. ss2-II). In this way, Figure 3 depicts the results from the immunoassays of each sample/sample site on a heat map (number of positive hits and relative abundance), where differences regarding detectable biomarkers in relation to phylogenetic groups can be seen and provide useful information about the microbial community. Table S1 in Supplementary Materials lists the antibodies in the LDChip, those detected in these positive immunodetections, the source and the immunogen of each one, and their cluster, phylum, and reference.

The results revealed a high heterogeneity of the different samples and sites. In some cases, even in the same site, heterogeneity was observed between samples from different locations, as was the case of Grᴂnvatn Lake and in the two sulfur springs in Regensburg. Notwithstanding, a common feature of all of them was that the main phylum detected by the antibodies, corresponding with the majority of the positive immunodetections, was Gammproteobacteria, which showed a wide presence in almost every sample except for Bou. I (Figure 3).

The highest detection in the number and diversity of biomarkers through the LDChip168 appeared in the SS1 sample from Iceland (Grᴂnvatn Lake) and in the sediments from the MSI sulfidic spring in Regensburg (Figure 2 and Figure 3). In contrast, the lowest detection of positive signals, regarding number and diversity, was found in sample site 1 from Kaunertal Glacier (G.SS1), in the water sample from Sippenauer Moore sulfidic spring (RG.SMw), and even in the sediments of this sulfidic spring (RG.SMs). Though the hypersaline environment (Boulby) showed lower intensities than the rest of the samples in terms of relative fluorescence, the number of biomarkers detected was higher (Figure 3). Furthermore, the strongest fluorescence intensities appeared in sample 1 from Iceland (IS.SS1), with the exception of peak 3 in RG.MSI, where the highest peaks corresponded to antibodies raised against the iron-sulfur reducers (peak 19), psychrophilic cultures (peak 53), mesophilic cultures (peak 79), archaea (peak 95), cyanobacteria (peaks 110 and 112) and proteins and peptides (peak 154) clusters.

Contrary to what one might initially expect due to the cold nature of the environment, positive signals for psycrophilic-source antibodies were rarely found in the glacier samples of the current study, but several positive immunodetections belonging to the mesophilic cluster were present. Within this mesophilic cluster, relatively significant phylum diversity could be appreciated (Figure 2, column f), especially in G.SS2 samples. In general, both sample sites from Kaunertal Glacier displayed low intensities in fluorescence signals compared with the rest, but they were still higher than those from Boulby.

Interestingly, apart from being the richest in terms of number and types of detectable biomarker signals, the immunopatterns of IS.SS1 (acidic lake) and RG.MSI (sulfidic spring) were notably similar, with the exception of antibody signals coming from proteins. These were present in the lake samples but were not traceable in the sulfur-spring sediment. Nevertheless, the intensities of the peaks in Regensburg samples were lower than those from Grænavatn Lake, except for peak 3, which corresponded to the antibody named A183 taken from *Leptospirillum ferroxidans*.

**Figure 4:**
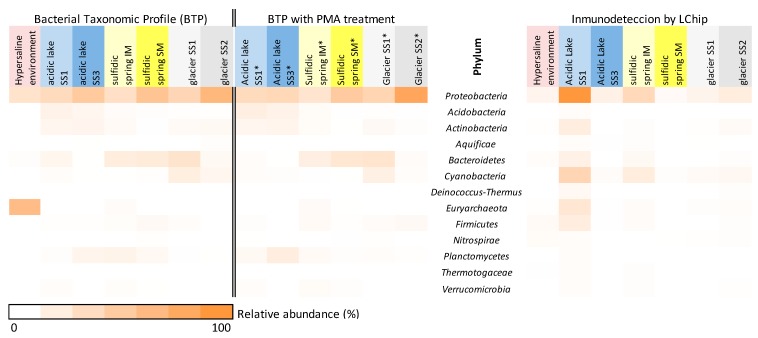
Comparison of bacterial abundance of the samples at phylum level. The heat maps display the relative abundance of phylum in each sample, where left panels show the universal primer set approach (samples without propidium monoazide (PMA) treatment and with PMA treatment-marked with asterisk*) and the right panel, the positive immunoreactions by the LDChip. Hypersaline environment: Boulby Mine (Bou); acidic lake SS1 and SS3 (Grænavatn Lake, IS.SS1 and IS.SS3); sulfidic spring IM and SM (Regensburg, RG.SM and RG.MSI); Glacier SS1 and SS2 (Kaunertal Glacier, G.SS1 and G.SS2). Sequences from hypersaline environment were not available for PMA treatment due to the interferences between salts and PMA [65].

As part of another study from MASE project about the microbial communities thriving in the MASE sampling sites [31] the German–Austrian group of the MASE team provided metagenomic data based on 16S rRNA gene amplicon and shotgun metagenome sequencing. The study was carried out by discerning sets of total bacterial and those living in the sample by the use of a DNA-intercalant: Propidium monoazide (PMA) that is a membrane-impermeant dye that intercalates into the DNA and distinguishes cells with compromised membranes from those intact (live) cells. [64]. Therefore, samples treated and untreated with PMA and subjected to metagenomics analysis by a universal primer set [31] showed some slight differences each other (Figure 4). Though these results, compared with the LDChip analysis (Figure 4), disclosed a moderate coincidence between relative abundance of sequences identified by metagenomics and the relative abundance of biomarkers detected, the high abundance of signals of Proteobacteria detected by both methods is still remarkable.

### 3.2. MASE-Chip

The LDChip results showed the presence of certain microbial groups and suggested different potential metabolic activities in the collected samples. Because of the inherent limitations of the LDChip due to its limited number of antibodies (168 in this case), we could not rule out the possibility of failing in the detection of a high number of microorganisms due to the absence of the appropriate antibody. Therefore, we contributed to enlarge antibodies collection for these Mars analog sites and prepared antibodies to a collection of 26 environmental isolates (Table 1) from the different MASE sites and from an existing antibodies’ collection from Molecular Ecology Laboratory (Centro de Astrobiologia-CAB, CSIC-INTA, Madrid). We tested and implemented them in a microarray chip, the so called MASE-Chip. We determined the optimal working concentration of the labelled antibodies and the specificity to assess the accuracy of the microarray following the procedures explained before in the Materials and Methods section. The working dilutions ranged from 1/800 to 1/900, and the lower detection limit of most of antibodies was established between 10^4^ and 10^3^ cells mL^−1^ (details of each antibody in Supplementary Materials, Table S2).

**Figure 5:**
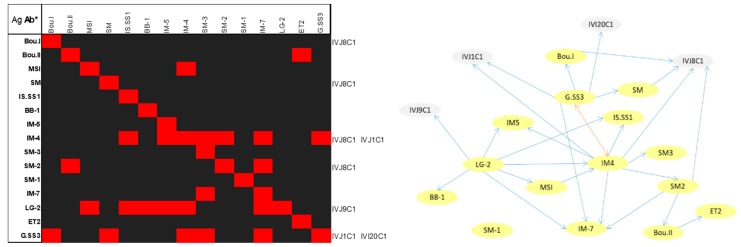
Cross-reactions in the MASE-chip. The heat map on the left shows the positive immunodetections (red squares) of the sandwich immunoassay *Ab capture-Ag-Ab trace* (*labelled). In the vertical left column, the tested antigens (so they are in rows) are represented. The top line of the table indicates Abs labelled (in columns). The outside column in the right part of the heat map shows the capture Abs printed on the array from previous collection and used in the building of MASE-Chip that cross-react and recognize MASE targets. The image on the right qualitatively shows the cross-reactivity events revealed in the heat map, where yellow circles represent the new Ab from MASE isolates, and the grey ones represent the Ab taken from previous studies. The blue arrows show the recognition of an Ab for the immunogen indicated. The orange arrow represents double direction recognition as happens between G.SS3 and IM-4 antibodies. The self-loops were not drawn to improve the clarity of the image. (The length of arrows is not informative of quantification).

From these analyses, we established the cross-reaction occurring in the MASE-Chip so that we could use this information to distinguish true antibody-target from other specific interactions due to shared antigens and discern false positives when analyzing a complex or environmental sample through it. These cross-reactions in the FSMI, and therefore the specificity of each Ab, were revealed by the positive immune detection measured and quantified through their relative fluorescence intensity in each case. The qualitative results are shown in Figure 5 as a heat map of the positive immunodetections and a diagram mapping the cross-reactions occurring in the MASE-Chip.

The results indicate that in several cases, the antibodies from the new MASE isolates were specific for their corresponding immunogen targets, although some were also captured by other antibodies on the microarray that came from a previous antibody collection. This occurred in the following cases: Bou.I, SM, IM-4 and SM-2—these immunogens also reacted with antibodies to *Halorubrum* sp. (IVJ8C1). Antibodies to *Haloferax mediterranei* (IVJ1C1) recognize IM-4 and G.SS3 antigens; antibodies to *Halobacterium* sp. (IVJ9C1) and to *Salinibacter ruber* M8 (IVI20C1) bound to LG-2 and G.SS3 immunogens, respectively. The highest cross-reactivity among the MASE antibodies identified was shown by LG-2, IM-4 and G.SS3. Noticeably, some Abs tested in the microarray recognized more than the one antigen that originated from them.

## 4. Discussion

Previous studies have extensively demonstrated the potentiality of LDChip technology, in its different versions, in several environments, such as the Atacama Desert, Glacial Lakes, acidic Rio Tinto River, permafrost in Antarctica, or deep South Africa Mines [40,47,48,62,67] as a reliable tool for environmental monitoring and profiling biomarkers and natural microbial communities present in ecosystems. Herein, we again demonstrated the potentiality and the ability of LDChip technology for a rapid assessment (a few hours) of the microbial profile of environmental crude samples in anoxic environments and its potential for biomarker detection in planetary exploration.

Regarding the studied biomarker profiles and taking into account not a phylogenetic criteria but a temperature tolerance as condition one, we identified a great absence of signals to antibodies from psychrophilic strains in contrast with the high number of those from mesophilic environments in the case of the glacier samples (Figure 2). The type of samples in this site, which were soil, could be prone to experiencing temperature fluctuation cycles to which mesophiles are better adapted [68]. In addition, although some studies have claimed that psychrophilic microorganisms are presumed to be predominant in those areas and niches permanently freeze [69], others have indicated the higher relative abundance of psychrotolerants and mesophiles over the amount of psychrophiles [70,71]. The absence of positive signals belonging to antibodies from psychrophilic bacteria in the samples of this study possibly responds to the fact that mesophilic biomass could be, as mentioned before, more abundant and could be better adapted than psychrophiles, causing a relative screening effect in biomarker detection. Another contribution to these results is the nature of the LDChip, which, although presenting a relatively similar number of antibodies of each group (19 from psychrophiles and 27 for mesophiles), detected antigenic determinants that could be shared by psychrophiles and mesophiles that were genetically related. Nevertheless, in glacier sample site 2 (G.SS2), biomarkers, of which sources were the psycrophiles *Psychrobacter cryohalolentis* (IVF8C1, peak 54 in immunogram Figure 2) and *Colwellia psychrerythraea* (IVF7C1, peak 53) were still perceptible, and the last one is considered as a facultative anaerobe that seems to adapt to cold environments by the production of extracellular polysaccharides [72].

In addition, among the samples, IS.SS1 from Grænavatn Lake displayed a large number of different biomarkers and presented the highest intensities in their fluorescence signals, where almost all phyla available in the LDChip were represented. Diversity data from MASE team studies on microbial community in MASE sampling sites [31], although with lower relative abundance and intensities, have been quite consistent with these findings and have shown a reasonable range of bacterial composition in sample IS.SS1 using a universal primer approach. Samples from Grænavatn Lake, especially IS.SS1, showed the prevalence of signals of antibodies from sources of metal-acidic environment, iron oxidizers cultures and acidophilic strains clusters that indicate the presence of this kind of molecular biomarkers or microorganisms from these groups or both [40]. The findings are relevant to this acidic lake, which is subject to periodic desiccation and has volcanic rock–water interactions [53]. These characteristics, in general, occur in the anoxic environments of Iceland and constitute sustained bodies of liquid water interacting with volcanic bedrock and with few other rock types represented; thus, these could be a potential analog for early Martian environments [73].

Regarding the sampled sulfidic springs sites (Regensburg), the RG.MSI sample seemed richer than the samples from RG.SM, which was probably due to the retention effect of sediments. These sediments retain organic compounds and nutrients, acting as a filter for water and, at the same time, constituting a nurturing enriched matrix that can serve as a favorable niche for microbes [74,75]. In this way, the water, apart from being a natural diluent of nutrients by itself, in these sites, is sieved by the sediments, which could explain the lower detection of biomarker signals in number and intensity.

Positive signals from those antibodies against proteins and peptides were more strongly detected in the salty environment and in the acidic lake in Iceland. This could provide clues about the kind of metabolism that could be occurring in the niche involving nitrogen and sulfate reduction (NRA, Prot_ApsA_RB11754, Prot_DsrA_RB11365, Prot_DsrB_RB11368, NirS, NOR1), synthesis of ATP (ASB, ASF1) and iron metabolism (Prot_FeReTs_983, Prot-Pfu-FER), among others (IDs of Abs in parentheses, see Table S1). Recent works using LDChip technology [43] have reported the presence of a rich microbial community in the shallow sediments of the lake shore of glacial lakes which may constitute a variety of anaerobic metabolisms. These kinds of metabolisms could have been occurring in the anoxic sites of this study in which similar positive signals have been detected by the immunoassays.

In the particular case of hypersaline samples, although there was a qualitative correlation in terms of some phylum clusters detected (Euryarcheaota, Bacterioidetes, and Proteobacteria) between biomarker and biodiversity data, some phylum which were also detectable by the chip were not identified by metagenomics (Actinobacteria, Firmicutes and Nitrospirae). It was observable that the proportion of each one in those detected by both approaches stayed different and that the positive signals obtained from microarray seemed lower especially for Proteobacteria. This deviation may have been caused by a disturbance over experimental procedures of the high salt concentration in samples, even though assay protocols were optimized to minimize these effects. Nevertheless, although the “salty-effect” caused a decrease in the intensity of the signals detected by the LDChip, the phylogenetic diversity of the biomarkers detected by this method remained higher. There was a relative bias regarding the nature of the antibodies printed in the LDChip168 due to different proportions of each group represented and because it did not cover the whole microbial diversity (Table S1). This could be the reason for the certain lack of correlation with DNA data, not only in these samples but also in the rest of the samples of this study (Figure 4). In addition, it is worth noting that the MASE site samples were subjected to microscopy examinations by the MASE team, which revealed a low amount of microbial cells, as well as abundant particles [31]. This probably caused certain metagenomic biases regarding DNA extraction and the sequencing process. It would be the responsible for certain deviations in data correlation between the LDChip and metagenomics in some samples regarding the less abundant phylums. This did not sufficiently affect in the case of the Proteobacteria phylum, members of which were widely identified by sequencing, and biomarkers from sources of it (46 antibodies among the total 168 antibodies in the LDChip) were detected by the LDChip for all the samples studied.

Nevertheless, taking data in combination, there was a grade of correspondence and mainly a complementation in the diversity of phylum in the Boulby samples and in the rest of the MASE sites, as well as the diversity in biomarkers detected by immunoassays. This, together with previous studies in which correlations between these techniques were relatively higher than in the MASE sites but still not absolutely coincident [40,43], supports the microarray approach as a reliable tool for biomolecules detection. Moreover, it drives the continuation of the development of specific microarrays for distinctive and particular features of environments using antibodies from sources of those features and adaptations. 

In general, based on the comparison of the two molecular approaches, the metagenomics analysis, and immunoassays with the LDChip, we could conclude, once again, that the LDChip is a highly useful immunosensing technique for biomarker and microbial detection which successfully complements and adds to the information obtained by classical techniques and with a desirable potential for planetary exploration. The power of this technique was recently proven for Mars exploration in a work which reported a remaining structural conformation of the molecules in the microarray enough for a successful recognition of epitopes under UV-radiation conditions relevant for Mars [76]. Our results contribute to enlarge the knowledge about the kind of potentially detectable biomarkers on Mars analog sites specifically and in others planetary aims in general.

Taking into account these supportive data and previous studies of the accuracy of a microarray approach for biomolecules detection in environment, a specific MASE-sites microarray was been designed and developed—MASE-Chip—to expand the contribution to the biomarkers detection field.

In the reported MASE-Chip, for the detection and identification of a wide range of biomolecules associated with halophiles and anaerobic, we used polyclonal antibodies. They have some advantages because they are cheaper and faster to produce and because the possibilities for binding any target epitope in a complex sample are theoretically higher than with monoclonal antibodies, especially in a sandwich type assay. The drawback of this selection is the apparently higher cross-reactivity between antigens in the use of polyclonal antibodies. To minimize this handicap, we selected high-titer antibodies, optimized working dilutions in the assays (Table S2), and disentangled cross-reaction occurring in the new array by the transformation of the fluorescence data into a matrix, which was visualized as a heat map and in a graph of nodes and links (Figure 5). Being aware of and taking into account what the heatmap and graph show can help the interpretation of data, in order to discern possible false positives or detection of similar microorganisms or metabolisms. Notwithstanding that in many cases, Abs tested in the microarray recognized more than the one antigen that originated from them (Figure 5), some phylogenetically related and others not, which responded to the mentioned cross-reactivity drawback that happens in multiplexed sandwich assays [77]. The circles and arrows in the graph presented in Figure 5 qualitatively show the events of recognition between Abs in the MASE-chip and their Ag cognates that were used for their performance. An improvement that will be implemented (and which has proven potential) will be to transform these data into quantitative data [78], which will help us to discard the weakest cross-reactions and keep those relevant.

The MASE-Chip is available to be used in future investigations, both in the field and laboratories in the search for molecular biosignatures. Further studies and improvements will support its accurate applicability for environmental and astrobiology research and, especially due to the nature of the sources used to generate the antibodies that it contains, in anaerobic and salty environments.

## Figures and Tables

**Table 1:** Antibodies printed in the MASE-Chip with their IDs (left column), their immunogen source, and their references (up: Unpublished). The first 15 of them came from isolates of MASE project and the rest belong to the Molecular Ecology Laboratory antibody collection at CAB.

Ab Name/ID	Immunogen (Sample/Strain)	References
Bou. I	Boulby Mine I (wet and pink salt)	-
Bou. II	Boulby Mine II	-
MSIs	Mülbach Islinger-Regensburg (cold spring-sediment)	-
SMs	Sippenauer Moore-Regensburg (cold spring-sediment)	-
IS. SS1	Grænavatn Lake-Iceland (sediment)	-
MASE-BB-1	*Halanaerobium* sp. (isolate from Boulby)	[30]
MASE-IM-5	*Trichococcus* sp. 37AN3 (Mülbach Islinger)	[30]
MASE-IM-4	*Clostridium* sp. DSM632 (Mülbach Islinger)	[30]
MASE-SM-3	*Hafnia* sp. (Sippenauer Moore)	[30]
MASE-SM-2	*Clostridium* sp. (Sippenauer Moore)	[30]
MASE-SM-1	*Methanomethylovorans* sp. (Sippenauer Moore)	[30]
MASE-IM-7	*Desulfovibrio* sp. (Mülbach Islinger)	[30]
MASE-LG-2	*Pelosinus* sp. (Grænavatn Lake)	[30]
ET2	*Bacteroides xylanoliticus* X5-1 (Grænavatn Lake)	up
MASE-Glacier-SS3	*Rhanella* sp. (Kaunertal Glacier)	[30]
IVE7C1	*Halothiobacillus neapolitanus*	[40]
IVG5C1	*Sulfobacillus acidophilus*	[40]
IVI12C1	*Geobacter metallireducens*	[62]
IVI20C1	*Salinibacter ruber M8*	[40]
IVI21C1	*Salinibacter ruber PR1*	[40]
IVI24C1	*Thessaracoccus lapidicapta*	[66]
IVJ1C1	*Haloferax mediterranei*	[62]
IVJ8C1	*Halorubrum* sp.	[40]
IVJ9C1	*Halobacterium* sp.	[40]
IVK19C1	*Chroococcidiopsis* O29	up
VD2BF	Biofilm from Mansimongs Mines Southafrica	up

## Supplementary Materials

The following are available online at https://www.mdpi.com/2076-2607/7/9/365/s1, Table S1: List of antibodies printed in the LDChip168 for this study. Table S2: Working dilution and detection limit of Abs in MASE-chip.
