# Supplementary material for: Microbial Markers Profile in Anaerobic Mars Analogue Environments Using the LDChip (Life Detector Chip) Antibody Microarray Core of the SOLID (Signs of Life Detector) Platform
Source: Microorganisms. 2019 Sep 18;7(9):365. doi: 10.3390/microorganisms7090365 (PMC6780502; doi:10.3390/microorganisms7090365)
Supplement: Supplementary file 1 [file microorganisms-07-00365-s001.pdf]

Peak	**Antibody name**	**Immunogen/ Antigen type**	**Source/Strain**	**Phylum**	**Group/Cluster**	**Positive in this study**	**Supp. References**
1	A139	Sonicated cells	*Leptospirillum ferroxidans*	Nitrospirae	Fe-S oxidizers cultures		[1]
2	IVE3C_182	Intact cells	*Acidithiobacillus ferroxidans*	Gammaproteobacteria	Fe-S oxidizers cultures	+	[1]
3	A183	Sonicated cells	*A. ferroxidans*	Gammaproteobacteria	Fe-S oxidizers cultures	+	[1]
4	A184	Intact cells	*A. ferroxidans*	Gammaproteobacteria	Fe-S oxidizers cultures	+	[1]
5	A186	Intact cells	*L. ferroxidans*	Nitrospirae	Fe-S oxidizers cultures	+	[1]
6	IVE1BF	Biofilm	*L. pherrifilum* (LPH2) fermentor	Nitrospirae	Fe-S oxidizers cultures	+	[2]
7	IVE1S100	Soluble cellular fraction (SB-S100)	*L. pherrifilum* (LPH2) fermentor	Nitrospirae	Fe-S oxidizers cultures	+	[2]
8	IVE1C2	Pellet insoluble cellular fraction	*L. pherrifilum* (LPH2) fermentor	Nitrospirae	Fe-S oxidizers cultures	+	[2]
9	IVE2S1	Supernatant	NtD (*Leptospirillum ferrifilum*)	Nitrospirae	Fe-S oxidizers cultures	+	[2]
10	IVE3C1	Celulas Intactas	*Acidithiobacillus caldus*	Gammaproteobacteria	Fe-S oxidizers cultures	+	[2]
11	IVE3S100	Soluble cellular fraction (SB-100)	*A. ferroxidans*	Gammaproteobacteria	Fe-S oxidizers cultures	+	[2]
12	IVE4C1	Intact cells	*Acidithiobacillus thioxidans*	Gammaproteobacteria	Fe-S oxidizers cultures	+	[2]
13	IVE4C2	Pellet S100	*A. thioxidans*	Gammaproteobacteria	Fe-S oxidizers cultures	+	[2]
14	IVE4S100	Soluble cellular fraction (SB-100)	*A. thioxidans*	Gammaproteobacteria	Fe-S oxidizers cultures	+	[2]
15	IVE5C1	Intact cells	*Acidithiobacillus albertensis*	Gammaproteobacteria	Fe-S oxidizers cultures	+	[2]
16	IVE6C1	Intact cells	*A. caldus*	Gammaproteobacteria	Fe-S oxidizers cultures	+	[2]
17	IVE6C2	Pellet S100	*A. caldus*	Gammaproteobacteria	Fe-S oxidizers cultures	+	[2]
18	IVE6S100	Soluble cellular fraction (SB-100)	*A. caldus*	Gammaproteobacteria	Fe-S oxidizers cultures		[2]
19	IVE7C1	Intact cells	*Halothiobacillus neapolitanus*	Gammaproteobacteria	Fe-S oxidizers cultures	+	[3]
20	IVE8C1	Intact cells	*Acidimicrobium ferrooxidans*	Actinobacteria	Fe-S oxidizers cultures	+	[3]
21	IVE9C1	Intact cells	*L. ferroxidans*	Nitrospirae	Fe-S oxidizers cultures	+	[4]
22	IA2C1	Intact cells	Green filaments from Río Tinto water	Cellular extract from environmental samples	Metal-acidic environ	+	[2]
23	IA3C1	Intact cells	Dark filaments from Río Tinto (Arroyo 3.1)	Cellular extract from environmental samples	Metal-acidic environ	+	[2]
24	IC10S1_141	First wash	Ground sediment	Extracellular substances from environmental samples	Metal-acidic environ	+	

25	IC1C1	Nycodenz	Beach lake 3,2	Cellular extract from environmental samples	Metal-acidic environ	+	[2]
26	IC3C1	Nycodenz	Source of Rio Tinto. Filaments	Cellular extract from environmental samples	Metal-acidic environ	+	[2]
27	IC3C3	Sonicated cells EDTA (Nycodenz)	Source of Rio Tinto. Filaments	Cellular extract from environmental samples	Metal-acidic environ	+	[2]
28	IC4C1	Nycodenz	Filaments from water dam 3,2	Cellular extract from environmental samples	Metal-acidic environ	+	[2]
29	IC4S2	Supernatant from EDTA wash	Filaments from water dam 3,3	Extracellular substances from environmental samples	Metal-acidic environ	+	[2]
30	IC6C1	Nycodenz	Red wet sediment 2cm deep (Playa 3,1)	Cellular extract from environmental samples	Metal-acidic environ	+	[2]
31	IC7C1	Nycodenz	Dry wall 3,2 seco	Cellular extract from environmental samples	Metal-acidic environ	+	[2]
32	ID17C1	Nycodenz	Fe-S precipitate from del 3,2. Río Tinto.	Cellular extract from environmental samples	Metal-acidic environ	+	[2]
33	ID18S2	Supernatant from EDTA	Peña de Hierro (93m deep) MARTE project	Extracellular substances from environmental samples	Metal-acidic environ	+	[2]
34	ID4S2	Supernatant from EDTA wash	Baton 61a, MARTE project	Extracellular substances from environmental samples	Metal-acidic environ	+	[2]
35	IIC1C1	Whole extract	Iceland sediment	Cellular extract from environmental samples	Geothermal environment	+	
36	IVF11C1	Intact cells	*Micrococcus*, strain Eur19.1	Actinobacteria	Psycrophilic cultures	+	
37	IVF13C1	Intact cells	*Frondihabitans*, strain AH2.2.5	Actinobacteria	Psycrophilic cultures		
38	IVF18C1	Intact cells	*Desulfotalea psychrophila*	Deltaproteobacteria	Psycrophilic cultures	+	[5]
39	IVF19C1	Intact cells	*Polaromonas*, strain Eur3.PT.21	Betaproteobacteria	Psycrophilic cultures	+	
40	IVF20C1	Intact cells	*Sphingobacteriu*, strain Eur3.AL.31	Bacteroidetes	Psycrophilic cultures		
41	IVF21C1	Intact cells	*Planomicrobium*, strain CY-C1-12	Firmicutes	Psycrophilic cultures	+	
42	IVF22C1	Intact cells	*Tumebacillus*, strain Eur1 9.5	Firmicutes	Psycrophilic cultures	+	
43	IVF23C1	Intact cells	*Paenibacillus*, strain Eur1 9.26	Firmicutes	Psycrophilic cultures	+	
44	IVF2C1	Intact cells	*Shewanella gelidimarina*	Gammaproteobacteria	Psycrophilic cultures	+	[2]
45	IVF2S100	Soluble cellular fraction (SB-100)	*S. gelidimarina*	Gammaproteobacteria	Psycrophilic cultures	+	[2]
46	IVF2S2	Supernatant from EDTA wash	*S. gelidimarina*	Gammaproteobacteria	Psycrophilic cultures	+	[2]
47	IVF31C1	Intact cells	*Planococcus or2*	Firmicutes	Psycrophilic cultures		[2]

48	IVF4C1	Intact cells	*Psychroserpens burtonensis*	Bacteroidetes	Psycrophilic cultures	+	[2]
49	IVF4S1	Supernatant	*P. burtonensis*	Bacteroidetes	Psycrophilic cultures	+	[2]
50	IVF4S2	SB del lavado de EDTA	*P. burtonensis*	Bacteroidetes	Psycrophilic cultures	+	[2]
51	IVF5C1	Intact cells	*Psychrobacter frigidicola*	Gammaproteobacteria	Psycrophilic cultures	+	[2]
52	IVF6C1	Intact cells	*Cryobacterium psychrophilum*	Actinobacteria	Psycrophilic cultures	+	[2]
53	IVF7C1	Intact cells	*Colwellia psychrerythraea*	Gammaproteobacteria	Psycrophilic cultures	+	[3]
54	IVF8C1	Intact cells	*Psychrobacter cryohalolentis*	Gammaproteobacteria	Psycrophilic cultures	+	
55	IVG1C1	Intact cells	*Acidocella aminolytica*	Alphaproteobacteria	Iron reducers	+	[3]
56	IVG2C1	Intact cells	*Acidiphillium* sp.	Alphaproteobacteria	Iron reducers	+	[3]
57	IVG3C1	Intact cells	*Acidobacterium capsulatum*	Acidobacteria	Iron reducers	+	[3]
58	IVG4C1	Intact cells	*Thermus scotoductus*	Deinococcus-Thermus	Iron reducers	+	[3]
59	IVG4C2	Pellet S100	*T. scotoductus*	Deinococcus-Thermus	Iron reducers	+	[3]
60	IVG5C1	Intact cells	*Sulfobacillus acidophilus*	Firmicutes	Iron reducers	+	[3]
61	IVH11C1	Intact cells	*Bacillus subtilis*	Firmicutes	Iron reducers		[4]
62	IVH1C1	Intact cells	*B. subtilis* (sonicated spores)	Firmicutes	Spores	+	[4]
63	IVH22C1	Intact cells	*Streptomyces* spores	Actinobacteria	Spores		[4]
64	IVH2C1	Intact cells	*Streptomyces* spores	Actinobacteria	Spores	+	
65	IVI10C1	Intact cells	*Desulfovibrio vulgaris subsp. Vulgaris*	Deltaproteobacteria	Mesophilic cultures	+	[2]
66	IVI11C1	Intact cells	*Geobacter sulfurreducens*	Deltaproteobacteria	Mesophilic cultures		[2]
67	IVI12C1	Intact cells	*Geobacter metallireducens*	Deltaproteobacteria	Mesophilic cultures	+	[2]
68	IVI13C1	Intact cells	*Thermotoga maritima*	Thermotogaceae	Mesophilic cultures	+	[2]
69	IVI14C1	Intact cells	*Verrucomicrobium spinosum*	Verrucomicrobia	Mesophilic cultures	+	[2]
70	IVI15C1	Intact cells	*Methylomicrobium capsulatum*	Gammaproteobacteria	Mesophilic cultures	+	[2]
71	IVI16C1	Intact cells	*Planctomyces limnophilus*	Planctomycetes	Mesophilic cultures	+	[2]
72	IVI17C1	Intact cells	*Hydrogenobacter thermophilus*	Aquificae	Mesophilic cultures	+	[2]
73	IVI19C1	Intact cells	*Desulfosporosinus meridiei*	Firmicutes	Mesophilic cultures	+	[3]
74	IVI1C1	Intact cells	*Pseudomonas putida*	Gammaproteobacteria	Mesophilic cultures	+	[2]
75	IVI20C1	Intact cells	*Salinibacter ruber* M8	Bacteroidetes	Mesophilic cultures	+	[3]

76	IVI21C1	Intact cells	*S. ruber* PR1	Bacteroidetes	Mesophilic cultures	+	[3]
77	IVI21C2	Pellet S100	*S. ruber* PR2	Bacteroidetes	Mesophilic cultures	+	[3]
78	IVI22C1	Intact cells	Isolated from Atacama	..	Mesophilic cultures		[4]
79	IVI23C1	Intact cells	Lisated mycelium from *Streptomyces*	Actinobacteria	Mesophilic cultures	+	[4]
80	IVI24C1	Intact cells	*Thessaracoccus lapidicapta*	Actinobacteria	Mesophilic cultures	+	[4]
81	IVI25C1	Intact cells	*Clostridium*	Firmicutes	Mesophilic cultures		[4]
82	IVI2C1	Intact cells	*Bacillus* spp (Rio Tinto source)	Firmicutes	Mesophilic cultures		[2]
83	IVI3C1	Intact cells	*Shewanella oneidensis*	Gammaproteobacteria	Mesophilic cultures	+	[2]
84	IVI3C2	pellet S100	*S. oneidensis*	Gammaproteobacteria	Mesophilic cultures	+	[2]
85	IVI4C1	Intact cells	*Burkholderia furngorum*	Betaproteobacteria	Mesophilic cultures	+	[2]
86	IVI5C1	Intact cells	*S. oneidensis*	Gammaproteobacteria	Mesophilic cultures	+	[2]
87	IVI5S100	Soluble cellular fraction (SB-100)	*S. oneidensis*	Gammaproteobacteria	Mesophilic cultures	+	[2]
88	IVI6C3	Sonicated cells EDTA	*Azotobacter vinelandii*	Gammaproteobacteria	Mesophilic cultures	+	[2]
89	IVI7C1	Intact cells	*Bacillus subtilis* 3610	Firmicutes	Mesophilic cultures	+	[2]
90	IVI8C1	Intact cells	*B. subtilis* 3610	Firmicutes	Mesophilic cultures	+	[2]
91	IVI9C1	Intact cells	*Deinococcus radiodurans*	Deinococcus-Thermus	Mesophilic cultures	+	[2]
92	IVJ1C1	Intact cells	*Haloferax mediterranei*	Euryarchaeota	Archaea	+	[2]
93	IVJ2C1	Intact cells	*Methanococcoides burtonii*	Euryarchaeota	Archaea		[2]
94	IVJ3C1	Intact cells	*Thermoplasma acidophilum*	Euryarchaeota	Archaea	+	[2]
95	IVJ4C1	Intact cells	*Methanobacterium formicicum*	Euryarchaeota	Archaea	+	[2]
96	IVJ5C1	Intact cells	*Methanosarcina mazeii*	Euryarchaeota	Archaea	+	[2]
97	IVJ6C1	Intact cells	*Pyrococcus furiosus*	Euryarchaeota	Archaea	+	[3]
98	IVJ8C1	Intact cells	*Halorubrum* sp.	Euryarchaeota	Archaea	+	[3]
99	IVJ9C1	Intact cells	*Halobacterium* sp.	Euryarchaeota	Archaea	+	[3]
100	IVK10C1	Intact cells. Cellular extract.	*Leptolyngbya boryana*	Cyanobacteria	Cyanobacteria		[6]
101	IVK11C1	Intact cells. Cellular extract.	*Tolypothrix distorta*	Cyanobacteria	Cyanobacteria		[6]
102	IVK12C1	Intact cells. Cellular extract.	*Aphanizomenon aphanizomenoides*	Cyanobacteria	Cyanobacteria	+	[6]
103	IVK13C1	Intact cells. Cellular extract.	*Nostoc* Antártida 16	Cyanobacteria	Cyanobacteria		[6]

104	IVK14C1	Intact cells. Cellular extract.	*Anabaena* Antartica 39	Cyanobacteria	Cyanobacteria	+	[6]
105	IVK15C1	Intact cells. Cellular extract.	*Leptolyngbya* Antartica 39	Cyanobacteria	Cyanobacteria	+	[6]
106	IVK16C1	Intact cells. Cellular extract.	*Tolypothrix* 17	Cyanobacteria	Cyanobacteria		[6]
107	IVK17C1	Intact cells. Cellular extract.	*Plankthotrix*	Cyanobacteria	Cyanobacteria		[6]
108	IVK18C1	Intact cells. Cellular extract.	*Chroococcidiopsis* O29	Cyanobacteria	Cyanobacteria	+	[4]
109	IVK19C1	Intact cells. Cellular extract.	*Chroococcidiopsis* O29	Cyanobacteria	Cyanobacteria	+	
110	IVK1C1	Intact cells. Cellular extract.	*Nostoc* grown in nitrate, *Anabaena* PCC7120	Cyanobacteria	Cyanobacteria	+	[6]
111	IVK1S2	Exopolisacarides fraction	*Nostoc* grown in nitrate, *Anabaena* PCC7120	Cyanobacteria	Cyanobacteria	+	[6]
112	IVK20C1	Intact cells. Cellular extract.	*Chroococcidiopsis* O57	Cyanobacteria	Cyanobacteria	+	
113	IVK21C1	Intact cells. Cellular extract.	*Chroococcidiopsis* O57	Cyanobacteria	Cyanobacteria	+	
114	IVK22C1	Intact cells. Cellular extract.	*Chroococcidiopsis* 171	Cyanobacteria	Cyanobacteria		
115	IVK2C1	Intact cells. Cellular extract.	*Nostoc* grown without nitrate, *Anabaena* PCC7120	Cyanobacteria	Cyanobacteria	+	[6]
116	IVK2S2	Exopolisacarides fraction	*Nostoc* grown without nitrate, *Anabaena* PCC7120	Cyanobacteria	Cyanobacteria	+	[6]
117	IVK3C1	Intact cells. Cellular extract.	*Microcystis flos-aquae*	Cyanobacteria	Cyanobacteria		[6]
118	IVK4C1	Intact cells. Cellular extract.	*Microcystis novacekii*	Cyanobacteria	Cyanobacteria	+	[6]
119	IVK5C1	Intact cells. Cellular extract.	*Microcystis aeruginosa*	Cyanobacteria	Cyanobacteria		[6]
120	IVK6C1	Intact cells. Cellular extract.	*Aphanizomenon ovalisporum*	Cyanobacteria	Cyanobacteria		[6]
121	IVK7C1	Intact cells. Cellular extract	*Phormidium* BGU3	Cyanobacteria	Cyanobacteria	+	[6]
122	IVK8C1	Intact cells. Cellular extract	*Rivularia* sp. MU15	Cyanobacteria	Cyanobacteria	+	[6]
123	IVK9C1	Intact cells. Cellular extract	*Chamaesiphon*	Cyanobacteria	Cyanobacteria		[6]
124	IVL10C1	Intact cells. Cellular extract	*Dechloromarinus chlorophilus*, strain NSS	Gammaproteobacteria	Perchlorate reducers		
125	IVL11C1	Intact cells. Cellular extract.	*Dechloromonas aromatica*, strain RCB	Betaproteobacteria	Perchlorate reducers		
126	IVL12C1	Intact cells. Cellular extract	*Arcobacter sp*., strain CAB	Epsilonproteobacteria	Perchlorate reducers	+	[4]
127	IVL1C1	Intact cells. Cellular extract	*Azospira suillum*, strain PS	Betaproteobacteria	Perchlorate reducers		
128	IVL2C1	Intact cells. Cellular extract	*Magnetospirillum bellicus*, strain VDY	Alphaproteobacteria	Perchlorate reducers		
129	IVL3C1	Intact cells. Cellular extract	*Ideonella dechloratans*	Betaproteobacteria	Perchlorate reducers		

130	IVL4C1	Intact cells. Cellular extract	*Dechlorobacter hydrogenophilus*, strain LT-1	Betaproteobacteria	Perchlorate reducers		
131	IVL5C1	Intact cells. Cellular extract	*Propionivibrio militaris*, strain MP	Betaproteobacteria	Perchlorate reducers	+	[4]
132	IVL6C1	Intact cells. Cellular extract	*Dechloromonas agitata*, strain CKB	Betaproteobacteria	Perchlorate reducers		
133	IVL6S2	Culture supernatant with ispropanol	*D. agitata*, strain CKB	Betaproteobacteria	Perchlorate reducers		
134	IVL7C1	Intact cells. Cellular extract	*Magnetospirillum* sp., strain WD	Alphaproteobacteria	Perchlorate reducers		
135	IVL8C1	Intact cells. Cellular extract	*Azospira* sp., strain ZAP	Betaproteobacteria	Perchlorate reducers		
136	IVL9C1	Intact cells. Cellular extract	*Shewanella algae*	Gammaproteobacteria	Perchlorate reducers		
137	VIIIA1V1	Virus *Salinibacter ruber* concentrated	*S. ruber*	Bacteroidetes	Halophilic viruses		[4]
138	VIID1BF	Whole extract (Guanidinio.HCl)	Environmental sample1 (gypsum curb / sulfates)	Environmental sample	Mesophilic environment	+	
139	VD2BF	Whole extract	Biofilm from Mansimongs mines (SA7 + SA9) South Africa	Biofilm from environmental samples	Mines	+	
140	ASB	ATP synthase, subunit B/Purified recombinant polipetide	*Archaeoglobus fulgidus*	Euryarchaeota	Proteins and peptides	+	[3]
141	ASF1	ATP synthase F1, subunit alpha/Purified recombinant polipetide	*Thermotoga maritima*	Thermotogaceae	Proteins and peptides	+	[3]
142	cld	Alpha-chlorite dismutase	*Dechloromonas agitata* strain CKB	Betaproteobacteria	Proteins and peptides	+	[7]
143	NRA	Nitrate reductase, alpha subunit/Purified recombinant polipetide	*Geobacter metallireducens*	Deltaproteobacteria	Proteins and peptides	+	[3]
144	Prot_PCR	Perchlorate reductase	*Dechloromonas aromatica*	Betaproteobacteria	Proteins and peptides	+	[4]
145	Prot_ABCtransporter	ABC transporter for nitrogenase (purified recombinant polypeptide)	*T. scotoductus*	Gammaproteobacteria	Proteins and peptides	+	[5]
146	Prot_ApsA_RB11754	Adenylylsulfate reductase alpha subunit	*Desulfovibrio desulfuricans*	Deltaproteobacteria	Proteins and peptides	+	[3]
147	Prot_DsrA_RB11365	Sulfite reductase, dissimilatory-type subunit alpha	*A. fulgidus*	Euryarchaeota	Proteins and peptides	+	[3]
148	Prot_DsrB_RB11368	Sulfite reductase, dissimilatory-type subunit beta	*A. fulgidus*	Euryarchaeota	Proteins and peptides	+	[3]
149	Prot_EFG_RB11359	Elongation factor G	*T. maritima*	Thermotogaceae	Proteins and peptides	+	[3]
150	Prot_ICDH_RB11756	Peroxisomal isocitrate dehydrogenase	*Arabidopsis thaliana*	Eukaryota (Viridiplantae)	Proteins and peptides	+	[3]
151	Prot_Lasparaginase	L-Asparaginase (purified)	*Escherichia coli* (Batch culture)	Gammaproteobacteria	Proteins and peptides	+	BIODESIGN (K59171R)

152	Prot_LBP+BSA	Lipopolysaccharide (in BSA)	*Pseudomonas sp.*	Gammaproteobacteria	Proteins and peptides	+	[3]
153	Prot_NADH_RB11364	NADH-quinone oxidoreductase subunit G	*Pseudomonas putida*	Gammaproteobacteria	Proteins and peptides	+	[3]
154	PCR A/B	Perchlorate reductase subunit alpha/beta	*Dechloromonas aromatica*	Betaproteobacteria	Proteins and peptides	+	[4]
155	Prot_FeReTs_983	Iron reductase/Purified recombinant polipetide	*T.scotoductus*	Gammaproteobacteria	Proteins and peptides	+	[3]
156	Prot-BaFER	Bacterioferritin	*D. desulfuricans*	Deltaproteobacteria	Proteins and peptides	+	[5]
157	Prot-LR1	ABC transporter (from South Africa mines)	*T. scotoductus*	Gammaproteobacteria	Proteins and peptides	+	[5]
158	Prot-Pfu-DPS	DNA protection during starvation protein	*P. furiosus*	Archaea	Proteins and peptides	+	[5]
159	Prot-Pfu-FER	Ferritin	*P. furiosus*	Archaea	Proteins and peptides	+	[5]
160	Prot-Sso-DPS	DNA protection during starvation protein	*Sulfolobus solfataricus*	Archaea	Proteins and peptides	+	[5]
161	RbcL	Ribulose bisphosphate carboxylase large chain	*A. thaliana*	Eukaryota (Viridiplantae)	Proteins and peptides	+	[3]
162	HtpG	Heat shock proteinG/Purified recombinant polipeptide	*Nostoc* PCC73102	Cyanobacteria	Proteins and peptides		[3]
163	HupS	Ni-Fe membrane hydrogenase S chain/purified recombinant polipeptide	*L. ferooxidans*	Nitrospirae	Proteins and peptides		[3]
164	McrB	Methyl CoM reductase I, B subunit	*Methanococcoides burtonii*	Euryarchaeota	Proteins and peptides		[3]
165	NifD	Nitrogenase protein alpha chain	*G. metallireducens*	Deltaproteobacteria	Proteins and peptides		[3]
166	NirS	Nitrite reductase	*Pseudomonas aeruginosa*	Gammaproteobacteria	Proteins and peptides		[3]
167	NOR1	Nitrite oxidoreductase Beta subunit	*Nitrobacter hamburgensis*	Alphaproteobacteria	Proteins and peptides		[3]
168	CspA	Cold shock protein A	*P. putida*	Gammaproteobacteria	Proteins and peptides		[3]

Table S1. List of antibodies printed in LDChip168 for this study. Please note that numbers in the first column (peak) correspond to the peak numbers in Fig. 2 and the positive sign (+) in the seventh column indicates those antibodies detected in one or more samples of this study.

Supplementary references

1. Parro, V.; Rodríguez-Manfredi, J.A.; Briones, C.; Compostizo, C.; Herrero, P.L.; Vez, E.; Sebastián, E.; Moreno-Paz, M.; García-Villadangos, M.; Fernández-Calvo, P., et al. Instrument development to search for biomarkers on mars: Terrestrial acidophile, iron-powered chemolithoautotrophic communities as model systems. Planetary and Space Science 2005, 53, 729-737, doi:https://doi.org/10.1016/j.pss.2005.02.003.
2. Rivas, L.A.; García-Villadangos, M.; Moreno-Paz, M.; Cruz-Gil, P.; Gómez-Elvira, J.; Parro, V. A 200-Antibody Microarray Biochip for Environmental Monitoring: Searching for Universal Microbial Biomarkers through Immunoprofiling. Analytical Chemistry 2008, 80, 7970-7979, doi:10.1021/ac8008093.
3. Parro, V.; Diego-Castilla, G.d.; Moreno-Paz, M.; Blanco, Y.; Cruz-Gil, P.; Rodríguez-Manfredi, J.A.; Fernández-Remolar, D.; Gómez, F.; Gómez, M.J.; Rivas, L.A., et al. A Microbial Oasis in the Hypersaline Atacama Subsurface Discovered by a Life Detector Chip: Implications for the Search for Life on Mars. 2011, 11, 969-996, doi:10.1089/ast.2011.0654.
4. Sánchez-García, L.; Aeppli, C.; Parro, V.; Fernández-Remolar, D.; García-Villadangos, M.; Chong-Diaz, G.; Blanco, Y.; Carrizo, D.J.B. Molecular biomarkers in the subsurface of the Salar Grande (Atacama, Chile) evaporitic deposits. 2018, 140, 31-52, doi:10.1007/s10533-018-0477-3.
5. Rivas, L.A.; Aguirre, J.; Blanco, Y.; González-Toril, E.; Parro, V. Graph-based deconvolution analysis of multiplex sandwich microarray immunoassays: applications for environmental monitoring. 2011, 13, 1421-1432, doi:doi:10.1111/j.1462-2920.2011.02442.x.
6. Blanco, Y.; Quesada, A.; Gallardo-Carreño, I.; Aguirre, J.; Parro, V. CYANOCHIP: An Antibody Microarray for High-Taxonomical-Resolution Cyanobacterial Monitoring. Environmental Science & Technology 2015, 49, 1611-1620, doi:10.1021/es5051106.
7. O'Connor, S.M.; Coates, J.D. Universal immunoprobe for (per)chlorate-reducing bacteria. Applied and environmental microbiology 2002, 68, 3108-3113, doi:10.1128/AEM.68.6.3108-3113.2002.

Ab Name/ID	Ab* (labelled) concentration (dilution factor from original [2 mg mL^{-1}])	Ag limit detection (cels/ml)
Bou. I	*1/900*	*1/1000**
Bou. II	*1/800*	*1/1**
MSIs	*1/800*	*1/100**
SMs	*1/800*	*1/100**
IS. SS1	*1/800*	*1/100**
MASE-BB-1	*1/800*	10^3
MASE-IM-5	*1/900*	10^3
MASE-IM-4	*1/800*	10^4
MASE-SM-3	*1/800*	10^4
MASE-SM-2	*1/800*	10^4
MASE-SM-1	*1/900*	10^3
MASE-IM-7	*1/800*	10^3
MASE-LG-2	*1/900*	10^3
ET2	*1/900*	10^4
MASE-Glacier-SS3	*1/800*	10^4

Table S2. Data from assays of new antibodies performed in the core of MASE project and printed as part of the MAS-Chips. First column refers to the working dilutions of antibodies ranged from 1/800 to 1/900 on the basis of the original concentration which is raised to 2 mg mL^{-1} after the purification of each one with Protein A (see materials and methods, section 2.3). The second columns shows the lower detection limit of antibodies that was established between 10^4 and 10^3 cells mL^{-1} in the antigenic sample (*it could not been established for the environmental samples in terms of number of cells mL^{-1} but was approximated by serial dilutions of the extracted antigen used to generate the corresponding Ab).
